# Resting energy expenditure of women with and without polycystic ovary syndrome: a systematic review and meta-analysis*

**DOI:** 10.64898/2025.12.03.25341536

**Published:** 2026-01-06

**Authors:** Richard Kirwan, Leigh Peele, Gregory Nuckols, Georgia Kohlhoff, Hannah Cabré, Alyssa Olenick, James Steele

**Affiliations:** 1 Research Institute for Sports and Exercise Sciences, Liverpool John Moores University, Liverpool, UK; 2 MacroFactor, Stronger by Science Technologies LLC, Raleigh, North Carolina, USA; 3 Flourishing Health, Liverpool, UK; 4 Reproductive Endocrinology and Women’s Health Research Program, Pennington Biomedical Research Center, Baton Rouge, Louisiana, USA; 5 Dr Alyssa Olenick LLC; 6 Steele Research Limited, Eastleigh, Hampshire, UK; 7 School of Health and Biomedical Sciences, Royal Melbourne Institute of Technology, Melbourne, VIC, Australia

**Keywords:** Polycystic ovary syndrome, Energy Metabolism, Resting Metabolic Rate, Basal Metabolism

## Abstract

**Context:**

Polycystic ovary syndrome (PCOS) is common in reproductive-age women, who often have higher BMI classification. This is assumed to stem from lower resting energy expenditure (REE), influencing lifestyle intervention guidelines. However, evidence for reduced REE in women with PCOS compared with those without is inconsistent.

**Objective:**

To systematically search and meta-analyse the existing literature to estimate and describe the difference in REE between women with and without PCOS.

**Data Sources:**

A systematic search was conducted using PubMed, Medline and Web of Science databases of published research from January 1990 to January 2025.

**Study Selection:**

Studies that measured REE in women living with PCOS, both with and without control arms of women without PCOS, were included.

**Data Extraction:**

Bibliometric, demographic, and REE data was extracted by one investigator and checked in triplicate.

**Data Synthesis:**

Thirteen studies were included in a Bayesian arm-based multiple condition comparison (i.e., network) type meta-analysis model with informative priors to compare both mean REE, and between person variation in REE, between women with and without PCOS. Mean REE differed between groups by 30 kcal/day [95% quantile interval: −47 to 113 kcal/day] and the contrast ratio for between person standard deviations was 0.98 [95% quantile interval: 0.71 to 1.33].

**Conclusions:**

These findings indicate that REE does not meaningfully differ between women with and without PCOS. Group-level differences in resting energy expenditure are small, insignificant, or not physiologically relevant.

## Introduction

Polycystic ovary syndrome (PCOS) affects approximately 10% of women of reproductive age worldwide, making it the most common endocrine disorder affecting this population^1^. Due to several factors including hyperandrogenism and alterations in insulin resistance, PCOS is believed to contribute to an increased risk of diabetes, metabolic syndrome and cardiovascular disease^2–6^, along with being a leading cause of anovulatory infertility in women^7^. Furthermore, epidemiological data has consistently demonstrated that women with PCOS are significantly more likely to suffer from overweight or obesity, compared to the general female population, with estimates ranging from 38% to 88% of PCOS patients falling into overweight or obese body mass index (BMI) categories^8,9^.

The elevated incidence of overweight and obesity in PCOS is likely multifactorial with proposed mechanisms including blunted postprandial appetite hormone responses leading to reduced satiety and increased food cravings^10–12^ and a reduced resting energy expenditure (REE)^13^. Indeed, the study from Georgopoulos et al.^13^ examined REE in women with and without PCOS using indirect calorimetry and reported that resting energy expenditure was approximately ~400 kcal/day lower in women with PCOS. Notably, they also reported that insulin resistance further reduced REE among women with PCOS, with insulin-resistant women exhibiting an additional reduction nearly 500 kcal per day compared to women with PCOS who were not insulin resistant^13^. Other studies similarly report lower REE in women with PCOS using indirect methods (such as prediction from bioelectrical impedance analysis or accelerometer physical activity data), also suggesting that factors including insulin resistance and BMI category influence REE in women with PCOS^14,15^. However, despite the widespread acceptance that women living with PCOS exhibit reduced REE based on studies such as these, other research has reported little to no difference in REE between women with and without PCOS^16–18^.

The consequences of widespread acceptance that REE is substantially lower in women living with PCOS should not be underestimated, particularly in light of the aforementioned incidence of overweight and obesity in this population. Women with PCOS typically engage in more frequent weight-loss attempts than women without PCOS^19^. From a physiological perspective, if women with PCOS do exhibit a lower REE, this could imply a meaningful metabolic disadvantage that may influence dietary and nutritional guidance for weight management; for example, recommending a slightly more severe energy restriction to overcome the belief that they have a lower REE^20^. Recommendations such as this could influence the well documented prevalence of eating disorders in women with PCOS^21,22^. Contrastingly, belief in a “slower metabolism” could instead serve as a deterrent to energy restriction based weight-loss approaches for some women in line with typical general population recommendations that are also recognised as efficacious for improving PCOS symptoms^23,24^ and are routinely recommended^25,26^. It has been well documented that women with PCOS already experience higher rates of anxiety, depression, and lower quality of life (QOL) as a result of negative body image and weight-related concerns^27–30^. Clarifying the relationship between REE and PCOS may therefore help guide more accurate clinical recommendations and empower both practitioners and women with PCOS.

A recent scoping review has highlighted the inconsistency in research examining energy expenditure in women with PCOS^31^. However, to our knowledge there has been no attempts to systematically review, and quantitatively synthesise via meta-analysis, studies of REE in women with, and without, PCOS. Therefore, to estimate and describe the magnitude of difference in REE between women with and without PCOS, we completed a systematic review and meta-analysis of studies reporting REE in these populations.

## Materials and Methods

**Figure 1:**
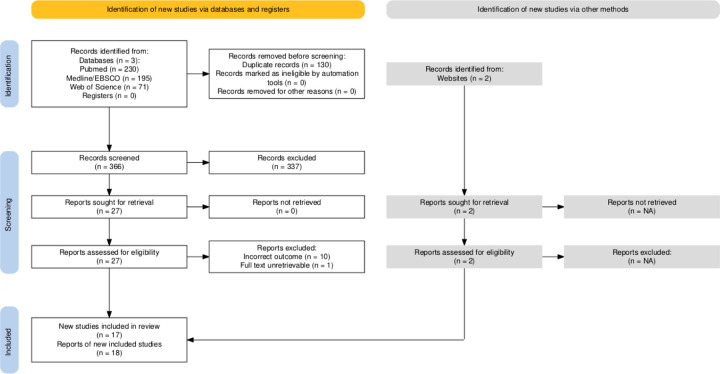
PRISMA 2020 flow diagram template for systematic reviews. Note that a “report” could be a journal article, preprint, conference abstract, study register entry, clinical study report, dissertation, unpublished manuscript, government report or any other document providing relevant information. The website noted was a prior narrative review on this topic by some of the authors (https://macrofactorapp.com/pcos-bmr/) which identified two studies not found in our systematic database search.

This systematic review and meta-analysis was pre-registered on PROSPERO (CRD42024601434) initially on the 3^*rd*^ of December 2024 and performed in accordance with the Preferred Reporting Items for Systematic Reviews and Meta-Analyses (PRISMA) statement guidelines^32^. The PRISMA flow diagram reported below (Figure 1) was produced using the PRISMA2020 R package and Shiny app^33^. The primary aim of this review was to examine the descriptive question “Does resting energy expenditure (REE) differ between women with and without polycystic ovary syndrome (PCOS)?”. We summarise and describe the studies in addition to quantitatively synthesising their results via meta-analysis.

### Search Strategy

PubMed, Web of Science, and MEDLINE databases were searched using the following Boolean search string: ((“Basal Metabolic Rate”[MeSH] OR “Energy Metabolism”[MeSH] OR “Resting Metabolic Rate” OR RMR OR “Resting Energy Expenditure” OR REE OR “Basal Metabolic Rate” OR BMR OR “resting energy” OR “basal energy expenditure”) AND (“Polycystic Ovary Syndrome”[MeSH] OR “Polycystic Ovary Syndrome” OR PCOS OR “Polycystic Ovarian Disease” OR “Stein-Leventhal Syndrome”)). Searches were limited to publications up until May 2025 when the search was completed, limited to English language articles, and Rayyan was used to manage the search and screening process. Two reviewers (RK and GK) independently screened all titles and abstracts against the predefined inclusion and exclusion criteria. Articles deemed potentially eligible by either reviewer were retrieved in full text. Full texts were then independently assessed by RK and LP to determine final eligibility. Any disagreements at either stage were resolved through discussion, and when consensus could not be reached, a third reviewer acted as an adjudicator.

### Eligibility Criteria

Studies were included in the systematic review if 1) participants were confirmed as women with PCOS between the ages of 18 to 65 years of age with or without insulin resistance; 2) otherwise healthy (e.g., non-diabetic, no cardiovascular disease); 3) had a measure of REE measured via multiple methods including direct/indirect calorimetry, doubly labelled water; and 4) trials were not retracted at the time of this analysis. Studies were excluded if they 1) used invalid or non-standard methods for measuring REE (e.g., predicted REE from body composition or accelerometer data); 2) non-peer-reviewed journal articles (including grey literature sources such as conference abstracts, theses and dissertations); and 3) were secondary analyses with the same primary outcome data as another included study.

The condition being studied was PCOS and we included observational cross-sectional design studies, in addition to intervention studies where REE was reported for the population (and if present, the comparator i.e., women without PCOS) condition of interest. For clarity, studies of any design were included if they reported the REE using the methods indicated for a sample of adult women with PCOS and who were otherwise healthy. This included both studies with and without samples of healthy control women without PCOS. As detailed in the statistical analysis section below, a Bayesian model with informative priors based on normative data for REE in healthy women without PCOS was included to provide control information indirectly where this was missing. The use of such priors is an efficient tool for incorporating historical information on a particular population in a conservative manner^34^.

Following the PICO framework our eligibility criteria can be defined as follows:

- Population
  - Inclusion criteria:
    - Women
    - 18–65 y
    - With or without insulin resistance (IR)
- Intervention(s) or exposure(s)
  - Otherwise healthy women with PCOS
- Comparator(s) or control(s)
  - Otherwise healthy control women without PCOS
- Outcome
  - Inclusion criteria
    - Resting energy expenditure (REE) measured via multiple methods including direct/indirect calorimetry, doubly labelled water.
  - Exclusion criteria:
    - Studies using invalid or non-standard methods for measuring REE (e.g., predicted REE from body composition or accelerometer data)

### Data extraction (selection and coding)

Data was extracted by one investigator and checked in triplicate. Bibliometric data including authors, journal, and article titles were extracted. Descriptive statistics for age, body mass, fat mass, fat free mass, height, BMI, race, physical activity levels, country of investigation, information regarding glucose/insulin regulation and insulin resistance status (where available), diagnostic criteria for PCOS, and measurement method and device were extracted for each arm within each study in addition to sample size. Descriptive characteristics were then tabulated across studies for reporting.

For each arm, and observation time point if multiple observations reported (e.g., before and after an intervention), depending on what was reported by the authors we extracted the means, medians, standard deviations, standard errors, lower and upper range values, and interquartile range for the unadjusted and/or body mass adjusted and/or fat free mass adjusted REE values. Where REE values adjusted for body mass and/or fat free mass were reported we used the reported body mass and/or fat free mass mean values for that arm to convert them to unadjusted REE values (i.e., multiplied them by body mass and/or fat free mass mean values). Where means and/or standard deviations were missing the latter were either calculated from standard errors and sample size, or all both were estimated from lower and upper range, interquartile range, median, and sample size depending on the available information using the methods of Wan et al.^35^. Further, where missing, height/body mass/BMI where estimated based on the reported means. The units of measurement for which REE was extracted and all REE values were converted to kcal/day. In one case^36^ REE was reported relative to body mass and the unadjusted values were no longer available (confirmed by the authors). As such, in this case we used the mean body mass to convert back to estimated REE unadjusted.

#### Studies with possible reporting errors

During data extraction it was noted that several studies from the same lab/research group^13,37–39^ contained a number of discrepancies that seemed to be possible reporting errors. This included, based on taking the authors results as written, standard errors that implied impossible or at least incredibly unlikely standard deviations, and discrepancies in sample size reporting throughout for most variables without explanation or where this was explained the sample sizes were discrepant with the text Further, data was not reported for the healthy control women without PCOS in three of the studies^37–39^, and REE was reported as an “adjusted” value whereby ${\text{REE}}_{adjusted}={\text{REE}}_{group\;\;mean}+\left({\text{REE}}_{adjusted}-{\text{REE}}_{predicted}\right)$ and the ${\text{REE}}_{predicted}$ was obtained by substituting the individual lean body mass, fat mass, gender, and age in the linear regression equation generated by the data of all patients. In correspondence with the senior author we were unable to clarify the reporting discrepancies as the person responsible for the data/results was no longer contactable. The original data were also no longer available and so we could not calculated the unadjusted REE.

Given these issues we decided to extract the results from these studies as reported and to conduct analyses both with and without their inclusion. Though not pre-registered, due to a lack of confidence in the reported results, we decided to include the analysis omitting these studies as our main models in the results reported below. The results of the analysis including them are reported in the sensitivity analysis section.

### Statistical Analysis

Statistical analysis of the data extracted was be performed in R, (v 4.3.3; R Core Team, https://www.r-project.org/) and RStudio (v 2023.06.1; Posit, https://posit.co/). All code utilised for data preparation, transformations, analyses, plotting, and reporting are available in the corresponding GitHub repository https://github.com/jamessteeleii/pcos_ree_meta. We cite all software and packages used in the analysis pipeline using the grateful package^40^ which can be seen here: https://github.com/jamessteeleii/pcos_ree_meta/blob/main/grateful-report.pdf. The statistical analysis plan was linked in our pre-registration (PROSPERO: CRD42024601434) and available at the accompanying GitHub repository. Any deviations from the pre-registration are noted below.

Given our research question our analysis was aimed at parameter estimation^41^ within a Bayesian meta-analytic framework^42^. For all analyses model parameter estimates and their precision, along with conclusions based upon them, are interpreted continuously and probabilistically, considering data quality, plausibility of effect, and previous literature, all within the context of each model. The renv package^43^ was used for package version reproducibility and a function based analysis pipeline using the targets package^44^ was employed (the analysis pipeline can be viewed by downloading the R Project and running the function targets::tar_visnetwork()). Effect sizes and their variances were all calculated using the metafor packages escalc() function^45^. The main package brms^46^ was used in fitting all the Bayesian meta-analysis models. Prior and posterior draws were taken using marginaleffects^47^ and tidybayes^48^ packages. All visualisations are created using ggplot2^49^, tidybayes, and the patchwork^50^ packages.

#### Main Pre-registered Models

We adopted an arm-based multiple condition comparison (i.e., network) type model given that the studies included had arms of women with PCOS both with, and without, a non-PCOS control arm^51^, and also in some cases multiple observations of REE in the different arms included in the study (for example, where an intervention was conducted and pre- and post-intervention REE was reported). In typical contrast-based meta-analyses data is limited to the effect sizes for paired contrasts between arms and thus studies that include both arms (i.e., relative effects between non-PCOS control vs PCOS arms); however, in arm-based analyses the data are the absolute effects within each arm and information is borrowed across studies to enable both within condition absolute, and between condition relative contrasts to be estimated. We made use of historical information regarding REE in healthy control women without PCOS by setting informative priors based on meta-analysis of large scale studies reporting normative data for REE in this population. This was included to provide indirect control information where it was missing from particular studies. The use of historical priors like this is an efficient tool to incorporate historical information about a particular population in a conservative manner in meta-analyses^34^. From this model we focus on reporting the global grand mean estimate for the fixed between condition relative contrast for non-PCOS control vs PCOS arms as our primary estimand of interest (i.e., $\beta_{1}$ in both mean and standard deviation models). We examined both raw mean REE (i.e., the absolute mean REE in kcals per day for each arm) in addition to the between person standard deviation in REE (i.e., the absolute standard deviation in REE in kcals per day for each arm). Both models were multilevel in that they included nested random intercepts for both study and arm within study. In addition, and in deviation from our pre-registration, we also included lab as a random intercept as in some cases we had multiple studies from the same lab or research group. Lastly, the inclusion of a random intercept for each effect size was accidentally omitted from our pre-registration, and so this is also included in the model.

#### Mean REE Model

The main model for mean REE with cond representing the condition (either control or PCOS) was as follows:

$${\hat{\theta }}_{ijkl}\sim 𝓝\left(\mu_{ijkl},\sigma_{ijkl}^{2}\right)$$

$$\mu_{ijkl}=\beta_{0}+\beta_{1}{\text{cond}}_{\left[ijkl\right]}+\alpha_{0,\text{lab}\left[i\right]}+\alpha_{1,\text{lab}\left[i\right]}\text{cond}+\alpha_{0,\text{study}\left[j\right]}+\alpha_{1,\text{study}\left[j\right]}\text{cond}+\alpha_{0,\text{arm}\left[k\right]}+\alpha_{0,\text{effect}\left[l\right]}$$

$$\left(\begin{array}{c} \alpha_{0,\text{lab}\left[i\right]}\\ \alpha_{1,\text{lab}\left[i\right]} \end{array}\right)\sim 𝓝\left(\left(\begin{array}{c} 0\\ 0 \end{array}\right),\Sigma_{\text{lab}}\right),\;\;\;\Sigma_{\text{lab}}=\left(\begin{array}{cc} \sigma_{0,\text{lab}}^{2} & \rho_{\text{lab}}\sigma_{0,\text{lab}}\sigma_{1,\text{lab}}\\ \rho_{\text{lab}}\sigma_{0,\text{lab}}\sigma_{1,\text{lab}} & \sigma_{1,\text{lab}}^{2} \end{array}\right),\;\;\text{for}\;\;\;\text{lab}\;\;\;\text{i}=1,\ldots ,\text{I}$$

$$\left(\begin{array}{c} \alpha_{0,\text{study}\left[j\right]}\\ \alpha_{1,\text{study}\left[j\right]} \end{array}\right)∼𝓝\left(\left(\begin{array}{c} 0\\ 0 \end{array}\right),\Sigma_{\text{study}}\right),\Sigma_{\text{study}}=\left(\begin{array}{cc} \sigma_{0,\text{study}}^{2} & \rho_{\text{study}}\sigma_{0,\text{study}}\sigma_{1,\text{study}}\\ \rho_{\text{study}}\sigma_{0,\text{study}}\sigma_{1,\text{study}} & \sigma_{1,\text{study}}^{2} \end{array}\right),\;\;\text{for}\;\;\text{study}\;\;\text{j}=1,\ldots ,\text{J}$$

$$\alpha_{0,\text{arm}\left[k\right]}\sim 𝓝\left(0,\sigma_{0,\text{arm}}^{2}\right),\;\;\;\text{for}\;\;\text{arm}\;\;\text{k}=1,\ldots ,\text{K}$$

$$\alpha_{0,\text{effect}\left[l\right]}\sim 𝓝\left(0,\sigma_{0,\text{effect}}^{2}\right),\;\;\;\text{for}\;\;\text{effect}\;\;\text{l}=1,\ldots ,\text{L}$$

where ${\hat{\theta }}_{ijkl}$ is the lth mean REE estimate from the kth arm, for the jth study, conducted by the ith lab and $\sigma_{ijkl}^{2}$ is the corresponding sampling error for that estimate. The random intercepts for the ith lab, jth study, kth arm, and lth mean REE estimate are $\alpha_{0,lab\left[i\right]}$, $\alpha_{0,study\left[j\right]}$, $\alpha_{0,arm\left[k\right]}$, and $\alpha_{0,effect\left[l\right]}$ respectively each with standard deviation of $\sigma_{0,lab\left[i\right]}^{2}$, $\sigma_{0,study\left[j\right]}^{2}$, $\sigma_{0,arm\left[k\right]}^{2}$, and $\sigma_{0,effect\left[l\right]}^{2}$. The parameter $\beta_{0}$ represents the fixed effect estimate of REE for control conditions and $\beta_{1}$ the fixed effect estimate for the offset from this for the PCOS conditions (i.e., the difference between conditions). The estimated offset was allowed to vary across both labs and studies each reflected by $\alpha_{1,lab\left[i\right]}$ and $\alpha_{1,study\left[j\right]}$ respectively, and these effects were also modelled as correlated with the corresponding random intercepts with covariance $\Sigma_{lab}$ and $\Sigma_{study}$, and ${\text{corr}}_{\text{lab}}$ and ${\text{corr}}_{\text{study}}$ correlation matrices.

The priors for this model were as follows:

$$\beta_{0}\sim \text{Studuent-}t\left(3,1441.81,84.56\right)$$

$$\beta_{1}\sim \text{Studuent-}t\left(3,0,200\right)$$

$$\begin{array}{l} \sigma_{0,\text{study}}^{2}\sim \text{Half-student-}t\left(3,149.89,82.91\right) \end{array}$$

$$\sigma_{0,\text{lab}}^{2},\sigma_{1,\text{lab}}^{2},\sigma_{1,\text{study}}^{2},\sigma_{0,\text{arm}}^{2},\sigma_{0,\text{effect}}^{2}\sim \text{Half-student-}t\left(3,0,112.4\right)$$

$${\text{corr}}_{\text{lab}}\sim \text{LKJ}\left(1\right)$$

$${\text{corr}}_{\text{study}}\sim \text{LKJ}\left(1\right)$$

where the prior for $\beta_{0}$, which corresponded to the model intercept and mean REE in the control condition, was set based on meta-analysis of the mean REEs for women from two large studies of healthy people^52,53^ though set with a conservative degrees of freedom for the Student-*t* distribution.The random intercept $\sigma_{0,study}^{2}$ was set similarly to this. The prior for the fixed effect $\beta_{1}$, reflecting the difference between control and PCOS conditions was set based on a wide range of possible values considering the minimum and and maximum values of the ranges reported in the two studies noted (i.e., 2492 – 908 = 1584). We then set a prior that permits values approximately across this range of values with the majority of it’s mass centred around zero. The remaining random effects were set based on the default weakly regularising priors for brms and scaled to the expected response values using a half-student-*t* distribution with 3 degrees of freedom and μ=0, and both correlation matrices ${\text{corr}}_{lab}$ and ${\text{corr}}_{study}$ were set with an LKJcorr(1) distribution.

#### Standard Deviation of REE Model

The main model for the standard deviation of REE with cond representing the condition (either control or PCOS) was as follows:

$$\log\left({\hat{\theta }}_{ijkl}\right)\sim 𝓝\left(\mu_{ijkl},\sigma_{ijkl}^{2}\right)$$

$$\mu_{ijkl}=\beta_{0}+\beta_{1}{\text{cond}}_{\left[ijkl\right]}+\beta_{2}{\tilde{m}}_{\left[ijkl\right]}+\alpha_{0,\text{lab}\left[i\right]}+\alpha_{1,\text{lab}\left[i\right]}\text{cond}+\alpha_{0,\text{study}\left[j\right]}+\alpha_{1,\text{study}\left[j\right]}\text{cond}+\alpha_{0,\text{arm}\left[k\right]}+\alpha_{0,effect\left[l\right]}$$

$$m=\log\left(y_{i,\text{mean},\left[\text{ijkl}\right]}\right)$$

$$\tilde{m}\sim 𝓝\left(m,\sigma_{\log\left(y_{i,\text{mean},\left[\text{ijkl}\right]}\right)}^{2}\right)$$

$$\left(\begin{array}{c} \alpha_{0,\text{lab}\left[i\right]}\\ \alpha_{1,\text{lab}\left[i\right]} \end{array}\right)\sim 𝓝\left(\left(\begin{array}{c} 0\\ 0 \end{array}\right),\Sigma_{\text{lab}}\right),\;\;\;\Sigma_{\text{lab}}=\left(\begin{array}{cc} \sigma_{0,\text{lab}}^{2} & \rho_{\text{lab}}\sigma_{0,\text{lab}}\sigma_{1,\text{lab}}\\ \rho_{\text{lab}}\sigma_{0,\text{lab}}\sigma_{1,\text{lab}} & \sigma_{1,\text{lab}}^{2} \end{array}\right),\;\;\text{for}\;\;\;\text{lab}\;\;\;\text{i}=1,\ldots ,\text{I}$$

$$\left(\begin{array}{c} \alpha_{0,\text{study}\left[j\right]}\\ \alpha_{1,\text{study}\left[j\right]} \end{array}\right)∼𝓝\left(\left(\begin{array}{c} 0\\ 0 \end{array}\right),\Sigma_{\text{study}}\right),\Sigma_{\text{study}}=\left(\begin{array}{cc} \sigma_{0,\text{study}}^{2} & \rho_{\text{study}}\sigma_{0,\text{study}}\sigma_{1,\text{study}}\\ \rho_{\text{study}}\sigma_{0,\text{study}}\sigma_{1,\text{study}} & \sigma_{1,\text{study}}^{2} \end{array}\right),\;\;\text{for}\;\;\text{study}\;\;\text{j}=1,\ldots ,\text{J}$$

$$\alpha_{0,\text{arm}\left[k\right]}\sim 𝓝\left(0,\sigma_{0,\text{arm}}^{2}\right),\;\;\;\text{for}\;\;\text{arm}\;\;\text{k}=1,\ldots ,\text{K}$$

$$\alpha_{0,\text{effect}\left[l\right]}\sim 𝓝\left(0,\sigma_{0,\text{effect}}^{2}\right),\;\;\;\text{for}\;\;\text{effect}\;\;\text{l}=1,\ldots ,\text{L}$$

where $\log\left({\hat{\theta }}_{ijkl}\right)$ is the lth natural logarithm of the standard deviation of REE estimate from the kth arm, for the jth study, conducted by the ith lab and $\sigma_{ijkl}^{2}$ is the corresponding sampling error for that estimate. The random intercepts for the ith lab, jth study, kth arm, and lth standard deviation of REE estimate are $\alpha_{0,lab\left[i\right]}$, $\alpha_{0,study\left[j\right]}$, $\alpha_{0,arm\left[k\right]}$, and $\alpha_{0,effect\left[l\right]}$ respectively each with standard deviation of $\sigma_{0,lab\left[i\right]}^{2}$, $\sigma_{0,study\left[j\right]}^{2}$, $\sigma_{0,arm\left[k\right]}^{2}$, and $\sigma_{0,effect\left[l\right]}^{2}$. The parameter $\beta_{0}$ represents the fixed effect estimate of standard deviation of REE for control conditions and $\beta_{1}$ the fixed effect estimate for the offset from this for the PCOS conditions (i.e., the difference between conditions). The estimated offset was allowed to vary across both labs and studies each reflected by $\alpha_{1,lab\left[i\right]}$ and $\alpha_{1,study\left[j\right]}$ respectively, and these effects were also modelled as correlated with the corresponding random intercepts with covariance $\Sigma_{lab}$ and $\Sigma_{study}$, and ${\text{corr}}_{\text{lab}}$ and ${\text{corr}}_{\text{study}}$ correlation matrices. Finally, $\beta_{2}$ represents the fixed effect of the natural logarithm of the corresponding mean REE estimate $\tilde{m}$ which is modelled as estimated with measurement error i.e., m represents the point estimate for the lth natural logarithm of the mean REE estimate from the kth arm, for the jth study, conducted by the ith lab and $\sigma_{\log\left(y_{i,\text{mean},\left[\text{ijkl}\right]}\right)}^{2}$ is the corresponding sampling error for that estimate. The priors for this model were as follows:

$$\beta_{0}\sim \text{Studuent-}t\left(3,5.54,0.80\right)$$

$$\beta_{1}\sim \text{Studuent-}t\left(3,0,5.3\right)$$

$$\beta_{2}\sim \text{Studuent-}t\left(3,0,2.5\right)$$

$$\text{mean}\left(\tilde{m}\right)\sim \text{Half-student-}t\left(3,7.28,0.62\right)$$

$$\text{sd}\left(\tilde{m}\right)\sim \text{Half-student-}t\left(3,0,5\right)$$

$$\sigma_{0,\text{study}}^{2}\sim \text{Half-student-}t\left(3,1.08,1.06\right)$$

$$\sigma_{0,\text{lab}}^{2},\sigma_{1,\text{lab}}^{2},\sigma_{1,\text{study}}^{2},\sigma_{0,\text{arm}}^{2},\sigma_{0,\text{effect}}^{2}\sim \text{Half-student-}t\left(3,0,2.5\right)$$

$${\text{corr}}_{\text{lab}}\sim \text{LKJ}\left(1\right)$$

$${\text{corr}}_{\text{study}}\sim \text{LKJ}\left(1\right)$$

where the prior for $\beta_{0}$, which corresponded to the model intercept and standard deviation of REE in the control condition, was again set based on meta-analysis of the standard deviation of REEs for women from two large studies of healthy people^52,53^ though set with a conservative degrees of freedom for the Student-*t* distribution.The random intercept $\sigma_{0,study}^{2}$ was set similarly to this. The prior for the fixed effect $\beta_{1}$, reflecting the difference between control and PCOS conditions was set based on a wide range of possible values. Given that in many cases of variables in the field there is an approximate relationship of ~1 for the natural logarithm of the standard deviation conditioned upon the natural logarithm of the mean^54^ we set this prior to reflect the range of differences on the on the log scale (i.e., $\log\left(1584\right)$). We then set a prior that permits values approximately across this range of values with the majority of it’s mass centred around zero. The prior for the fixed effect $\beta_{2}$, reflecting the relationship between the natural logarithm of the mean REE with the natural logarithm of the standard deviation of REE, was set it to be weakly informative centred on zero with a wide scale to indicate uncertainty in this outcome specifically despite the typical relationship close to ~1. Priors for the measurement error of the natural logarithm of the mean REE estimate $\tilde{m}$ were again based upon meta-analysis of the aforementioned studies, though measurement error has to be positive, as does the corresponding standard deviation of this error, so we set these to conservative wide half-student-*t* distributions. The remaining random effects were set based on the default weakly regularising priors for brms and scaled to the expected response values using a half-student-*t* distribution with 3 degrees of freedom and μ=0, and both correlation matrices ${\text{corr}}_{lab}$ and ${\text{corr}}_{study}$ were set with an LKJcorr(1) distribution.

#### Post-processing of models

For both models we examined trace plots along with $\hat{R}$ values to examine whether chains have converged, and posterior predictive checks for each model to understand the model implied distributions. From each model we took draws from the posterior distributions for the conditional absolute estimates for each condition (i.e., controls and PCOS) by study incorporating random effects, the global grand mean absolute estimates for each condition ignoring random effects, and the global grand mean between condition relative contrast for controls vs PCOS conditions ignoring random effects. The between condition relative contrast for controls vs PCOS conditions corresponded to $\beta_{1}$ in each model and was our primary estimand of interest; for the mean REE model this corresponded to the absolute difference in mean REE, and for the standard deviation of REE model this corresponded to the natural logarithm of the ratio of standard deviations of REE which was exponentiated (note, all log standard deviation of REE model estimates were exponentiated back to the original scale to aid interpretibility). We present the full probability density functions for posterior visually, and also to calculate mean and 95% quantile intervals (QI: i.e., ‘credible’ or ‘compatibility’ intervals) for each estimate providing the most probable value of the parameter in addition to the range from 2.5% to 97.5% percentiles given our priors and data.

#### Sensitivity analyses

##### Pairwise contrast based models

By way of pre-registered sensitivity analysis we also conducted pairwise contrast based models where we limited the included effects to those extracted from studies including only a directly comparable control and PCOS arm at baseline. These models were both as follows:

$${\hat{\theta }}_{ij}\sim 𝓝\left(\mu_{ij},\sigma_{ij}^{2}\right)$$

$$\mu_{ij}=\beta_{0}+\alpha_{0,\text{lab}\left[i\right]}+\alpha_{0,\text{study}\left[j\right]}$$

$$\alpha_{0,\text{lab}\left[i\right]}\sim 𝓝\left(0,\sigma_{0,\text{lab}}^{2}\right),\;\;\;\text{for}\;\;\text{lab}\;\;\text{i}=1,\ldots ,\text{I}$$

$$\alpha_{0,\text{study}\left[j\right]}\sim 𝓝\left(0,\sigma_{0,\text{study}}^{2}\right),\;\;\;\text{for}\;\;\text{study}\;\;\text{j}=1,\ldots ,\text{J}$$

where ${\hat{\theta }}_{ijkl}$ is the pairwise effect size, either the mean difference in REE or the log coefficient of variation ratio (calculated as PCOS vs control), for the jth study, conducted by the ith lab and $\sigma_{ijkl}^{2}$ is the corresponding sampling error for that effect size estimate. The random intercepts for the ith lab, jth study are $\alpha_{0,lab\left[i\right]}$ and $\alpha_{0,study\left[j\right]}$ respectively each with standard deviation of $\sigma_{0,lab\left[i\right]}^{2}$, $\sigma_{0,study\left[j\right]}^{2}$. The parameter $\beta_{0}$ represents the fixed effect estimate of the pairwise effect size i.e., the pooled estimate of the contrast between conditions. The priors for $\beta_{0}$ in these models were set as default weakly regularising which is set on an intercept that results when internally centering all population-level predictors around zero to improve sampling efficiency and scaled to the expected response values using a student-*t* distribution; for the mean difference in REE this was student-*t*(3, 5.5, 50.2) and for the log coefficient of variance ratio this was student-*t*(3, −0.1, 2.5). The random effects were set similarly scaled to the expected response values but using a half-student-*t* distribution centred on zero. From these models we calculated the mean and 95% quantile intervals (i.e., ‘credible’ or ‘compatibility’ intervals) for the $\beta_{0}$ (comparable to the $\beta_{1}$ from the corresponding mean and standard deviation of REE arm-based models) for each effect size providing the most probable value of the parameter in addition to the range from 2.5% to 97.5% percentiles given our priors and data.

#### Additional sensitivity analyses

As noted in the section above, *“Studies with possible reporting errors”*, we opted to conduct analysis with and without the inclusion of four studies with possible reporting errors we could not resolve^13,37–39^. Thus the main models described above were run with and without these studies, the main results are presented without them and the sensitivity results with them are reported separately below.

As a further sensitivity analysis, given the inclusion of some studies with interventions in women with PCOS reporting REE at multiple timepoints such as mid- and post-intervention^36,55,56^ but lacking of control women without PCOS, we opted to also conduct sensitivity analysis excluding these and only examining their baseline results in addition to the cross-sectional studies. As such, the main models described above were run without the follow-up (i.e., mid- or post-intervention timepoints) from these studies and only using the baseline results in addition to other cross-sectional studies.

Lastly, given the role of body mass and, in particular, highly metabolically active tissue on REE we included models adjusted for these group level characteristics where studies reported them. These models were essentially extensions of the main models noted above for BMI and fat-free mass where each was modelled with it’s corresponding sampling error similarly to how the log mean REE was modelled in the models for standard deviation of REE i.e., the mean BMI or fat-free mass estimates were modelled as estimated with measurement error. When extracting posterior distributions for the contrasts between conditions in these models both BMI and fat-free mass where adjusted to the median values seen in the control conditions i.e., BMI = 26.03 kg/m^2^ and fat-free mass = 48.8 kg.

## Results

### Systematic Review

Note, the numbers in this section exclude the four studies previously noted with possible reporting issues that were unresolved. These studies are however summarised in fully in the descriptive characteristics table in the online supplementary materials (see Descriptives Table where some of the possible reporting errors are seen in the standard deviations reported or calculated e.g., the standard deviation calculated for body mass of women with PCOS in Saltamavros et al.^38^. The PRISMA flow diagram (Figure 1) also includes all studies identified including the four with possible errors.

Our systematic review identified 13 studies from 12 lab/research groups including 24 arms (Control arms = 9, PCOS arms = 15) and a total of 918 participants (Controls: minimum n = 9, median n = 29, maximum n = 54; PCOS: minimum n = 5, median n = 28, maximum n = 266). Descriptive characteristics of the arms and participants in these studies are reported fully in the online supplementary materials (see Descriptives Table).

Studies were carried out in multiple countries: Brazil (k = 3) ,and USA (k = 3 studies), Australia, Cameroon, Canada, Italy, Sweden, Turkey, UK (all k = 1 study). The four studies with noted reporting errors were carried out in Greece. A total of 11 studies used the Rotterdam criteria (or a modified version there of) for diagnosing PCOS^16,17,36,55–62^. One study used the 1990 National Institutes of Health criteria^63^, and two studies^18,57^ diagnosed PCOS via the presence of oligomenorrhea or amenorrhea alongside additional criteria including plasma androgen levels, hirsutism or polycystic ovaries on ultrasound scanning. All but one study^63^, which used doubly labelled water, measured REE using indirect calorimetry. The specific devices reported by these studies are included in the online supplementary materials (see Descriptives Table). We originally considered in our pre-registration that, given sufficient data, we would compare sub groups of women with PCOS who did and did not have accompanying insulin resistance. However, based on the metabolic health variables reported in studies with this information (see Descriptives Table) and, where available, considering primary criteria of either homeostatic model assessment of insulin resistance (HOMA-IR) ≥ 2.5 or secondary criteria including fasting insulin > 12*μ*U/mL or fasting glucose ≥ 100mg/dL, all groups of women with PCOS in the included studies would be considered to have insulin resistance. Mean age of women with PCOS in these studies ranged from 23 to 33 which was similar to the control women without PCOS ranging from 23 to 30. Across those studies where BMI was reported or it was possible to estimate the mean BMI of women with PCOS ranged from 26.4 to 39.9 was typically greater than women without PCOS which ranged 20.5 to 27.9.

### Mean REE Model Results

**Figure 2:**
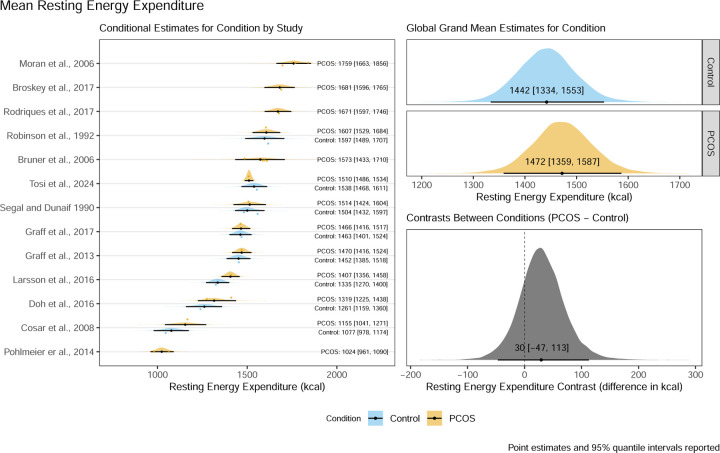
Posterior distribution, mean point estimates, and 95% quantile intervals for conditional estimates by study, global grand mean estimates by condition, and the contrast between conditions for mean resting energy expenditure of control women without PCOS and women with PCOS.

The main model for mean REE resulted in a posterior distribution for the contrast between control and PCOS conditions with a mean point estimate of 30 kcal/day with a 95% quantile interval ranging from −47 kcal/day to 113 kcal/day suggesting there is a 95% probability that the true difference lies between these values given our priors and the data from included studies. The corresponding conditional estimates for the control condition and PCOS condition respectively were 1442 kcal/day [95%QI:1334 kcal/day to 1553 kcal/day] and 1472 kcal/day [95%QI:1359 kcal/day to 1587 kcal/day]. These results, including the full visualisation of the posterior distribution and the conditional estimates by study, can be seen in Figure 2.

### Standard Deviation of REE Model Results

**Figure 3:**
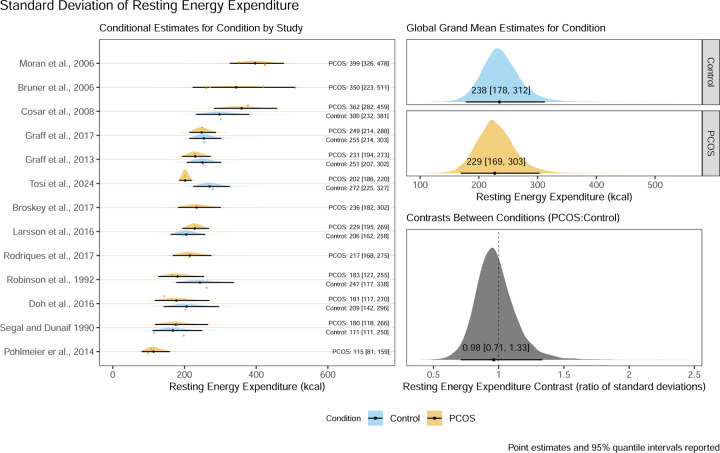
Posterior distribution, variance point estimates, and 95% quantile intervals for conditional estimates by study, global grand variance estimates by condition, and the contrast ratio between conditions for the standard deviation of resting energy expenditure of control women without PCOS and women with PCOS.

The main model for the between participant standard deviation of REE resulted in a posterior distribution for the contrast ratio between control and PCOS conditions with a mean point estimate of 0.98 with a 95% quantile interval ranging from 0.71 to 1.33 suggesting there is a 95% probability that the true ratio of standard deviations lies between these values given our priors and the data from included studies. The corresponding conditional estimates for the standard deviations of the control condition and PCOS condition respectively were 238 kcal/day [95%QI:178 kcal/day to 312 kcal/day] and 229 kcal/day [95%QI:169 kcal/day to 303 kcal/day]. These results, including the full visualisation of the posterior distribution and the conditional estimates by study, can be seen in Figure 3.

### Sensitivity Analyses

#### Pairwise Models

For mean REE the pairwise model resulted in qualitatively similar inferences suggesting little difference between control and PCOS conditions with mean point estimate of 16 kcal/day with a 95% quantile interval ranging from −36 kcal/day to 70 kcal/day. This was similar for the standard deviation of REE with the pairwise model resulting in a contrast ratio between the control and PCOS conditions with a mean point estimate of 0.9 and a 95% quantile interval ranging from 0.6 to 1.35.

#### Models including studies with possible reporting issues

For mean REE the model including the studies noted with possible reporting issues that were not resolved^13,37–39^ still resulted in qualitatively similar inferences suggesting little difference between control and PCOS conditions with mean point estimate of 15 kcal/day with a 95% quantile interval ranging from −69 kcal/day to 95 kcal/day. This was similar for the standard deviation of REE with the model including these studies resulting in a contrast ratio between the control and PCOS conditions with a mean point estimate of 1.21 and a 95% quantile interval ranging from 0.68 to 2.13.

#### Basline REE Measurement Models

For mean REE the model which included only baseline REE measurements from studies involving interventions^36,55,56^, in addition to other cross-sectional studies, also resulted in qualitatively similar inferences suggesting little difference between control and PCOS conditions with mean point estimate of 32 kcal/day with a 95% quantile interval ranging from −46 kcal/day to 118 kcal/day. This was also the case for the standard deviation of REE with this model resulting in a contrast ratio between the control and PCOS conditions with a mean point estimate of 0.97 and a 95% quantile interval ranging from 0.71 to 1.36.

#### BMI and Fat-Free Mass Adjusted Models

For mean REE the model adjusted for BMI, also resulted in qualitatively similar inferences suggesting little difference between control and PCOS conditions with mean point estimate of 20 kcal/day with a 95% quantile interval ranging from −96 kcal/day to 140 kcal/day. This was also the case for the standard deviation of REE with this model resulting in a contrast ratio between the control and PCOS conditions with a mean point estimate of 0.91 and a 95% quantile interval ranging from 0.56 to 1.41. This was similar for fat-free mass adjusted models too showing little difference in mean REE between control and PCOS conditions with mean point estimate of −9 kcal/day with a 95% quantile interval ranging from −195 kcal/day to 168 kcal/day and a contrast ratio between the control and PCOS conditions with a mean point estimate of 0.79 and a 95% quantile interval ranging from 0.31 to 1.94.

## Discussion

This study sought to estimate and describe the magnitude of difference in REE between women with and without PCOS. Most studies identified in the systematic review, and included in the meta-analysis, used indirect calorimetry as the primary measure of REE and assessed women with PCOS who were insulin resistant and categorised as being in overweight or obese BMI categories compared to healthy controls. Our results indicate there is only a small magnitude of difference in REE (30 kcal/day [95%QI: −47 kcal/day to 113kcal/day]) between women with PCOS and those without. Further, there is little difference in between person variation between the groups based on the ratio of standard deviations (0.98 [95%QI: 0.71 to 1.33]) suggesting that, despite individual differences in REE, PCOS is not systematically associated with lesser or greater individual variability.

These findings challenge the widely held belief that PCOS is inherently associated with a slower metabolism^26^, predisposing women with PCOS to weight gain. This belief largely stems from a single but influential 2009 study from Georgopoulos et al. that reported a significantly reduced BMR in women with PCOS^13^, which has been widely cited and reinforced in both academic and clinical contexts. However, as we have noted, this study along with others^37–39^ have numerous reporting errors which led us to drop them from our present analysis (though sensitivity analysis including them did not alter our overall conclusions). The mistaken belief that REE is lower for those with PCOS may have mistakenly lead to recommendations centred on slightly more severe calorie restriction to achieve weight-loss goals, compared with recommendations for the general population, as primary management strategies for women with PCOS^20^. Recognising that there may be minimal differences in REE between women with PCOS and those without can inform both clinical and public practices, potentially leading to a shift in focus away from a requirement for more severe caloric restriction as a primary method of treatment towards more comprehensive, individualised, and psychologically safe approaches to care [moranEvidenceSummariesRecommendations2020;^26^].

The current study suggests that REE may not a barrier to weight regulation in PCOS given small group-level differences between women with PCOS and those without (1472 kcal/day [95%QI:1359 kcal/day to 1587 kcal/day] versus 1442 kcal/day [95%QI:1334 kcal/day to 1553 kcal/day], respectively). If anything, REE may be slightly higher in women with PCOS compared to healthy women without PCOS. BMI of women with PCOS ranged from 26.4 to 39.9 was typically greater than women without PCOS which ranged 20.5 to 27.9 and this may explain the slightly greater REE in the former group. However, for those studies where we could extract or estimate BMI, our additional exploratory models adjusted for this similarly showed little difference in REE (20 kcal/day [95%QI: −96 kcal/day to 140kcal/day]) between women with PCOS and those without. However, one of the studies included in our analysis^64^ reported that, whilst there was little difference in unadjusted REE, when REE was adjusted for fat-free mass it was lower in women with PCOS suggesting the potential importance of fat-free tissue in energy regulation. Yet, for those studies where fat-free mass was reported, our additional exploratory models adjusted for this similarly showed little difference in REE (−9 kcal/day [95%QI: −195 kcal/day to 168kcal/day]) between women with PCOS and those without. As such, even adjusted for both BMI and fat-free mass, there seems to be little difference in REE between women with, and without, PCOS. Yet, a recent systematic review of mechanisms for metabolic dysfunction has reported excess androgen drives metabolic issues within adipose tissue and muscle tissue contributing to complications like obesity and insulin resistance^65^. Taken together, these findings highlight that factors beyond REE and typical correlates of this including BMI or fat-free mass, such as hormonal and tissue-specific metabolic effects, may play a more significant role in weight regulation challenges in women with PCOS.

As noted, women with PCOS are more likely to engage in weight-loss attempts^19^ and there could be concerns that this could inadvertently further foster the already well documented disordered eating in this population^21,22,66,67^. The pathways linking PCOS and disordered eating are multifactorial. Biological mechanisms such as hyperandrogenism, hyperinsulinaemia, and altered ghrelin and leptin signalling can heighten hunger, carbohydrate cravings, and appetite variability^68^. Frequent hypoglycaemia and associated mood changes have also been observed, which can trigger compensatory eating or binge episodes^68^. These physiological processes interact with psychological and social stressors, including infertility concerns, conflicting nutrition advice, chronic dieting, and exposure to idealised body images on social media, which together compound vulnerability to disordered eating^69^. Moreover, eating disorders themselves can disrupt endocrine function, potentially worsening PCOS symptoms and creating a self-reinforcing cycle^67,70^.

Understanding the intertwined biological, psychological, and social influences suggests the importance of considering whether restrictive dietary advice is appropriate given it may exacerbate feelings of failure, hunger dysregulation, and shame^71^. These concerns, coupled with the lack of difference in REE between women with and without PCOS might suggest that energy restriction based dietary interventions for weight-loss may be unnecessary. But, there is also evidence supporting the effect of energy restricted dietary interventions for improving PCOS symptoms^23,24^ and they are recommended in international guidelines^25,26^. Encouragingly though, these guidelines also recognise weight stigma as a determinant of health and call for its reduction across clinical and public health settings. Evidently the greater prevalence of women with PCOS falling into overweight and obese BMI categories compared to women without PCOS^8,9^ is unlikely to be due to differences in REE and so, despite the potential effectiveness of energy restriction dietary interventions, there is potential value in moving towards more weight-neutral, individualised, and empowering care following holistic guidelines recommending multiple approaches to management^20,25,26^.

### Strengths and Limitations

The current study has multiple strengths stemming from its preregistered, comprehensive methodology and Bayesian statistical approach. This statistical framework allowed us to incorporate studies with and without control groups to better estimate REE in women with and without PCOS and to perform multiple sensitivity analyses that confirmed the stability of our findings. However, a limitation here is that variability in methods across studies, such as differences in PCOS diagnostic criteria and REE testing protocols, may have influenced results and the relatively small number of studies overall limits the extent to which we can explore these potential moderators. Furthermore, some studies controlled for body weight or body composition when reporting REE values, while others did not. We accounted for this by estimating or converting reported data to obtain unadjusted REE values across all groups, thereby reducing this variability and further as noted above provided estimates adjusted for BMI and fat-free mass both of which had little influence on our conclusions. Another limitation is that, due to fewer total control groups than PCOS groups, informed priors were required in several statistical models. However, in the context of Bayesian meta-analysis this can also be considered a strength. Additionally, some studies reported data inconsistencies that could not be clarified (e.g.,^13,37–39^) and were dropped from our main analysis though our conclusions again did not qualitative change when we conducted sensitivity analyses including these studies. Finally, most included studies were cross-sectional or baseline assessments within intervention trials, which limits causal inference. Indeed, we did not pre-register any kind of causal model (e.g., a directed acyclic graph) to inform our analysis approach for causal inference and as such have been explicit about the estimates presented as being descriptive.

## Conclusion

In conclusion, the findings from this meta-analysis indicate that REE does not meaningfully differ between women with and without PCOS. Group-level differences in REE were small, insignificant, or not physiologically relevant. Additionally, variability in REE between individuals was also similar. These results suggest that a lower baseline REE is not associated with the weight-related challenges often associated with PCOS. These findings challenge the popular narrative that women with PCOS have a lower REE and may help better inform dietary interventions and nutritional support for these individuals. Future research should include more standardized REE measurement and reporting protocols, greater data transparency, consistent control and reporting of body weight or body composition, the presentation of both absolute and relative REE, and more precise characterization of PCOS phenotypes. Overall, these findings support the conclusion that PCOS is not negatively associated with REE and may help practitioners and researchers focus on individually targeted and holistic lifestyle interventions rather than negatively framed interventions based on unsupported assumptions regarding REE.

## Data Availability

All code utilised for data preparation, transformations, analyses, plotting, and reporting are available in the corresponding GitHub repository https://github.com/jamessteeleii/pcos_ree_meta.
